# Decoding Infertility: the Epigenetic Influence of HDACs on Reproductive Function

**DOI:** 10.1007/s43032-026-02104-9

**Published:** 2026-04-24

**Authors:** Annanya Kapur, Yashaswini Reddy, Nanda Rajagopal, Shruthi Nayak, Neil Viren D’Souza, Ipshita Das, Babitha Kampa Sundara

**Affiliations:** Department of Biophysics, Manipal School of Life Sciences, Manipal Academy of Higher Education, Manipal, Karnataka 576104 India

**Keywords:** Epigenetics, HDACs, Infertility, Oogenesis, Spermatogenesis

## Abstract

Infertility is a multifactorial condition affecting approximately 10–15% of couples worldwide, with both male and female factors contributing equally. Among the emerging determinants of infertility are epigenetic regulators, particularly histone deacetylases, which modify chromatin structure and influence gene expression by removing acetyl groups from histone tails. Their dysregulation alters gene expression profiles critical for reproductive function. HDACs are classified into four major classes (I-IV) on the basis of their structure and function. A thorough analysis of previous research using animal models, clinical settings, and experiments was performed to investigate how HDACs function in gametogenesis, hormone regulation, and embryonic development, among other areas of reproductive physiology. HDACs play a key role in the epigenetic control of fertility in both sexes. Understanding their isoform-specific functions could help create more focused treatments for infertility and increase the success of reproduction. This review elucidates the mechanisms by which HDACs contribute to infertility and explores their potential as therapeutic targets in reproductive medicine.

## Introduction

Infertility is a disease, condition or status that is defined by the inability to achieve a successful pregnancy on the basis of the patient’s medical, sexual and reproductive history, age, physical findings, diagnostic testing, or any combination of those factors according to the American Society for Reproductive Medicine (ASRM) [1]. In accordance with WHO estimates published in 2023 based on data up to 2021, infertility affects approximately 17.5% of the adult population, or one in six people, at some point in their lives [2]. This suggests a significant, steady worldwide burden that affects both men and women across all income levels [3]. Recent studies highlight the crucial role of epigenetic control in reproductive health and disease, in addition to well-established genetic mutations, reproductive anatomical abnormalities, and endocrine dysfunctions [4]. Epigenetic modifications include heritable and reversible changes, such as DNA methylation, histone tail modifications, and noncoding RNAs, that dynamically influence gene expression without altering the DNA sequence [5]. Among epigenetic regulators, histone deacetylases (HDACs) are crucial enzymes that remove acetyl groups from lysine residues on histones and nonhistone proteins, resulting in chromatin condensation and transcriptional repression. This modulation has a significant effect on processes such as gametogenesis, folliculogenesis, embryo development, and implantation [6]. The cytoskeletal architecture and nonhistone substrates involved in signal transmission, which impact cellular differentiation and fertility-related functions, are also controlled by HDACs [7].

On the basis of their functional characteristics and sequence homology, HDACs in mammals can be divided into four main groups: Class I, Class II (IIa and IIb), Class III (sirtuins), and Class IV. Class I HDACs (HDAC1,2,3,8): These enzymes, which are present mostly in the nucleus, are widely expressed and play key roles in basic cellular functions such as cell division and proliferation, which are essential for gamete production and early embryogenesis [4]. Class IIa and IIb HDACs (HDAC 4, 5, 6, 7, 9, and 10): Class IIa enzymes move between the nucleus and cytoplasm and are involved in tissue-specific regulation, including endometrial receptivity and Sertoli cell activity. Members of class IIb, particularly HDAC6, are involved in sperm motility and target cytoskeletal proteins [8]. Class III HDACs (Sirtuins 1–7): Studies have connected SIRT1 and SIRT6 to oocyte quality and implantation outcomes, demonstrating the importance of these NAD⁺-dependent deacetylases in metabolic regulation and the oxidative stress response [9, 10]. Class IV HDACs (HDAC 11) share structural features with both class I and class II enzymes and have been implicated in immune regulation within the reproductive tract, although their reproductive functions continue to be poorly understood [11].

Recent functional investigations using a variety of model organisms have shown that important reproductive events are impacted by interruption or dysregulation of HDAC activity. HDAC1/2 deletion in oocytes in mouse models leads to follicular depletion and chromatin remodelling abnormalities, whereas uterine-specific HDAC3 depletion causes infertility due to uterine nonreceptivity and implantation failure [6, 12]. The evolutionary conservation of HDAC function in reproduction is shown by RNA interference (RNAi)-mediated silencing of HDAC genes, which reduces fertility and ovary development in insect models such as *Nilaparvata lugens* [13]. Environmental factors play a key role in epigenetic infertility. Endocrine disruptors alter HDAC expression and acetylation patterns in reproductive organs [14].

Assisted reproductive technologies (ARTs), including ovarian hyperstimulation, in vitro fertilisation (IVF), and embryo culture, may interfere with normal epigenetic programming during early embryogenesis [15]. These procedures expose gametes and embryos to nonphysiological hormonal environments and culture conditions that can disrupt the balance between histone acetyltransferases and histone deacetylases, thereby altering histone acetylation levels and chromatin accessibility [16]. Such disturbances may impair HDAC-mediated chromatin remodelling and transcriptional regulation during critical stages, such as zygotic genome activation, potentially leading to aberrant gene expression, imprinting defects, and compromised embryo development [17–19].

**Fig. 1:**
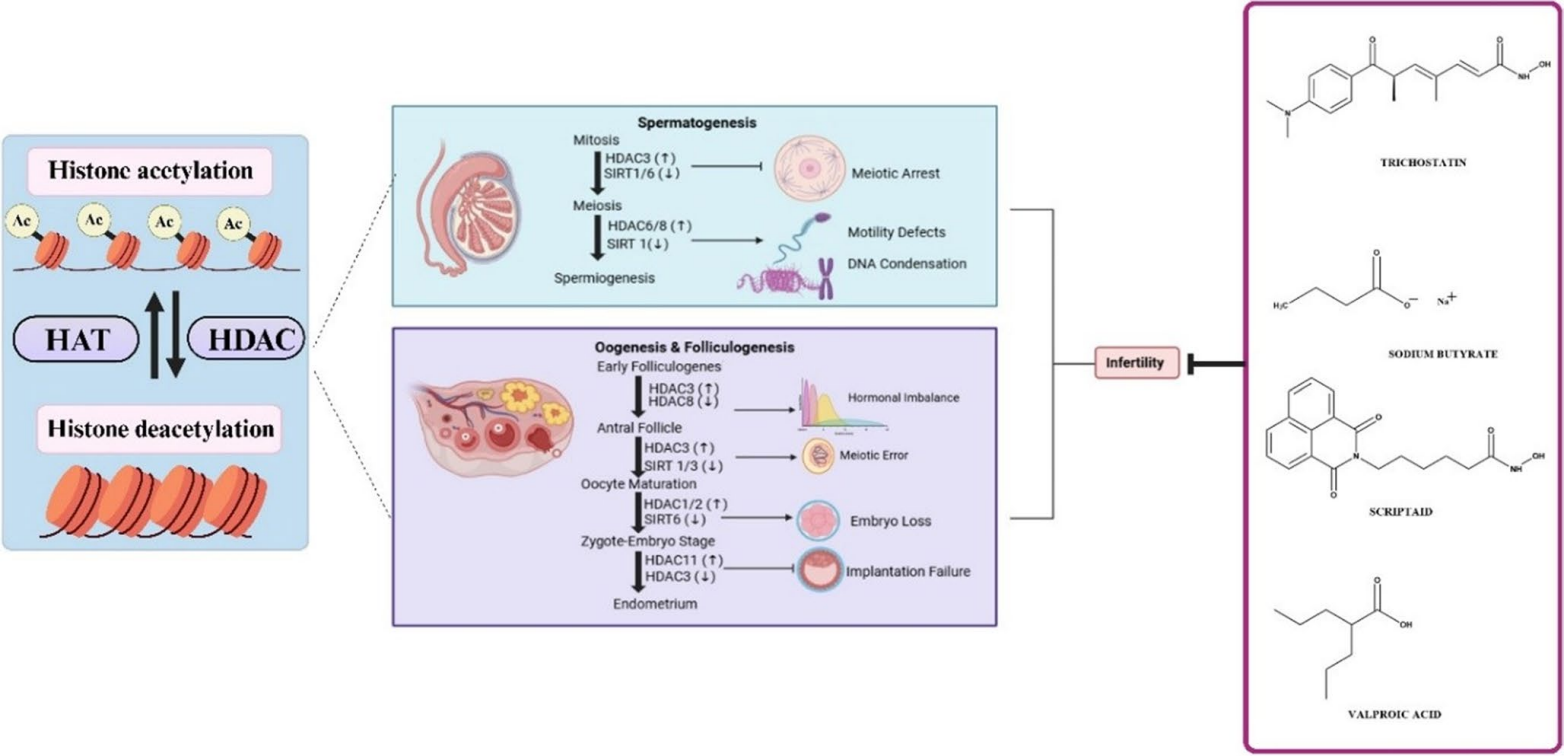
HDAC–HAT disequilibrium perturbs gametogenesis and early embryonic development, driving infertility and positioning HDACi as potential therapeutic modulators

In-depth knowledge of HDAC functions in reproductive genetics can possibly support the development of targeted infertility therapies such as sirtuin activators and isoform-selective HDAC inhibitors. This review summarises the current evidence concerning the role of HDACs in infertility from genetic, biochemical, pharmacological, and environmental viewpoints to inform future research and clinical translation (Fig. 1).

## HDAC Isoforms

### Class I HDACs

 Class I HDACs are expressed primarily in the nucleus and are widely expressed in germ cells as well as in gonad somatic cells [20]. Studies with oocyte-specific HDAC1/2-mutant mice have shown that these genes play important roles in preserving the chromatin architecture required for meiosis and preventing premature follicle loss [6]. Ma et al. [6] created oocyte-specific double knockout mice for HDAC1/2, which showed severe infertility due to faulty chromatin remodelling, spindle assembly, and transcriptional dysregulation, resulting in follicular atresia and halting early embryonic development. HDAC2 loss alone disrupts chromosomal segregation and embryo viability, emphasising isoform-specific activities [21]. Samartzis et al. [22] reported significantly elevated HDAC1 levels in the epithelium and stroma of human endometriotic lesions compared with those in eutopic endometrium from women without endometriosis. Positive correlations were observed between HDAC1 and estrogen/progesterone receptors (ER/PR), suggesting that class I HDACs play important roles in endometriosis pathogenesis [22]. Furthermore, HDAC1 and HDAC2 are essential during mouse preimplantation development for controlling chromatin remodelling, lineage commitment, and genome-wide DNA methylation through interactions with DNA methyltransferases (DNMTs), such as DNMT3A2. The loss of these genes disrupts epigenetic programming and embryonic cell differentiation, leading to developmental arrest and infertility [23].

In addition to HDAC1 and HDAC2, other class I HDACs, particularly HDAC3 and HDAC8, have emerged as critical regulators of reproductive function. The depletion of HDAC3 in mouse uterine stromal cells causes implantation failure by modifying decidualisation and progesterone signalling pathways, highlighting its role in endometrial receptivity [12]. Emerging evidence indicates that another class I enzyme, HDAC8, plays a significant role in reproductive physiology. In granulosa cells (GCs), HDAC8 regulates the expression of steroidogenic enzymes such as StAR and CYP19A1 as well as estradiol synthesis, indicating that it is involved in follicular maturation and hormone regulation [24]. In male reproduction, HDAC8 suppression in mouse testes impaired spermatogonial proliferation and Sertoli cell function, suggesting a role in testicular homeostasis. Aberrant HDAC8 expression is associated with endometriosis, and treatment with HDAC8 inhibitors in mice with induced deep endometriosis resulted in significantly improved pain behavior and reduced lesion weight [25]. At the functional level, selective class I histone deacetylase inhibitors (HDACis) affect four major aspects of zygotic genome activation (ZGA): RNA splicing, cell cycle regulation, autophagy, and transcription factor regulation. The class I-selective inhibitor MGCD0103 caused complete arrest at the 2-cell stage in mouse embryos *via* G2/M checkpoint disruption [26].

### Class II HDACs

Class IIa and IIb HDACs move between the nucleus and the cytoplasm, where they deacetylate histone and nonhistone substrates [27]. HDAC4 and HDAC5 form regulatory complexes with the DREAM protein to epigenetically regulate gene expression through coordinated deacetylation. This complex-mediated regulation has implications for developmental processes and cellular differentiation, including potential roles in germ cell development [28]. HDAC5, a class IIa HDAC, inhibits proinflammatory M1 polarisation of mouse testicular macrophages, which otherwise causes inflammation-mediated disruption of the blood‒testis barrier (BTB). The BTB is required to protect developing germ cells and ensure spermatogenesis. In mouse models, exposure to lipopolysaccharide (LPS) causes M1 polarisation and alters BTB function, increasing the likelihood of infertility. On the other hand, HDAC5 prevents macrophage-driven inflammation, protecting BTB integrity and testicular function [29].

HDAC6, known for its cytoplasmic localisation, regulates tubulin acetylation, which affects sperm motility in mouse models and meiotic spindle dynamics. HDAC6 knockout mice are alive and fertile under normal conditions but exhibit hyperacetylated tubulin and decreased sperm motility upon stress or pharmacological treatment. HDAC6 also plays an important role in spindle movement during oocyte meiosis, with pharmacological suppression resulting in an infertile phenotype [30]. HDAC6 is present in mouse testicular and caudal sperm, where it functions as an alpha-tubulin deacetylase. HDAC6 interacts with alpha-tubulin in sperm flagella and is catalytically active. Importantly, HDAC6 inhibitors (trichostatin A, tubastatin A, and sodium butyrate) increase acetyl alpha-tubulin acetylation and restrict sperm motility in mouse spermatozoa, indicating that HDAC6 is involved in modulating sperm movement and male fertility [31]. HDAC6 overexpression in GC-1 spg cells (mouse germ cells at the spermatogonial stage) significantly upregulated the expression of Profilin 2, a protein predominantly enriched in neurons but also detectable in germ cells. Profilin 2 colocalises with HDAC6 in both germ cells and mature sperm, suggesting that HDAC6 plays a role in the development of early germ cells and may influence spermatogenesis in addition to affecting sperm motility [32, 33].

Interestingly, pharmacological inhibition of HDAC6 has been demonstrated to improve oocyte maturation and sperm function in vitro in mouse gametes, suggesting that a mild reduction in HDAC6 expression can restore the acetylation equilibrium in gametes with abnormally high deacetylase activity [34]. As a result, HDAC6 functions as a regulator rather than a simple on-off switch, with both excessive and insufficient activity capable of compromising reproductive outcomes. HDAC6 also regulates primordial follicle activation through mTOR signalling in mice. The inhibition or knockdown of HDAC6 expression significantly promoted the activation of dormant primordial follicles while maintaining the total follicle pool. HDAC6 inhibition upregulated mTOR expression in mouse ovarian follicles and PI3K activity in oocytes, leading to the growth and differentiation of primordial follicle GCs, increased KITL secretion, and the awakening of dormant follicles in vitro [35].

The expression of class II HDACs, which are essential regulators of reproductive processes, is currently increasing, but class II HDACs have received far less academic attention than class I HDACs in the area of fertility. HDAC6 is the most widely researched class II HDAC because of its role in primordial follicle activation and sperm motility. The function of HDAC4 in oocyte maturation has received increased attention. HDAC5, HDAC7, and HDAC9 are underexplored in reproductive situations, indicating potential exploration for future research. Evidence from other organisms further supports the conserved role of HDACs in reproduction. For example, in insects such as the brown planthopper (*Nilaparvata lugens*), RNAi-mediated knockdown of NlHdac1, NlHdac3, and NlHdac4 disrupts ovary development, thereby causing female infertility and therefore leading to evolutionary conservation of HDAC function [13].

### Class III HDACs

Sirtuins (SIRT1-7) are NAD⁺-dependent deacetylases that regulate mitochondrial integrity, energy metabolism, and oxidative stress, which are necessary for gamete quality and follicular survival. SIRT1 and SIRT3 are important regulators of the female reproductive lifespan. These isoforms maintain oocyte quality and prolong ovarian aging in rodent models by regulating mitochondrial homeostasis and oxidative stress pathways. Both classical HDACs and sirtuins form epigenetic networks that regulate gamete competency and reproductive success. Functional studies in sirtuin-deficient animal models have revealed critical roles in fertility: knockout mice exhibit impaired folliculogenesis, increased oocyte apoptosis, and decreased fertility, whereas pharmacological sirtuin activation restores ovarian function and embryo developmental potential outcomes in mouse models [36]. The positive effects of resveratrol, which is a well-known sirtuin activator, highlight the therapeutic potential of regulating these enzymes. Resveratrol-mediated SIRT1 activation improved follicular reserve and reproductive outcomes in obese and metabolically stressed mice [37]. SIRT1 also protects GCs in vitro, and its knockdown accelerates oxidative stress-induced granulosa apoptosis through the SIRT1/p53 regulatory axis, demonstrating the critical role of SIRT1 in maintaining granulosa cell quality and preventing follicular atresia [38]. In aged mouse ovaries, SIRT1 upregulation improves granulosa cell function through increased PGC-1α-mediated mitochondrial biogenesis and a bidirectional regulatory mechanism involving insulin receptor substrate 1 (IRS1) in metabolic regulation and oxidative stress defense [39].

SIRT3 loss in mouse models causes reactive oxygen species (ROS) accumulation, follicular atresia, and impaired oocyte competence, which can be alleviated by low-oxygen culture or sirtuin-activating drugs. In contrast, SIRT4 expression is elevated during reproductive aging, indicating a compensatory but dysregulated metabolic response that affects oocyte competency [35]. SIRT6 and other sirtuins maintain telomere and genomic stability during the mouse preimplantation stage, hence ensuring reproductive success [40]. SIRT7 helps maintain genomic integrity by repairing double-strand breaks, which are essential for oocyte developmental competence. SIRT7-deficient mice exhibit lower embryonic viability and increased aging owing to the critical function of SIRT7 in DNA repair [41].

These studies provide insight into how class III HDACs (sirtuins) play critical roles in reproductive processes throughout the reproductive lifespan, ranging from hypothalamic control of the hypothalamic‒pituitary‒gonadal axis (HPG) to direct regulation of gametogenesis, embryo development, and ovarian aging. The most widely studied sirtuins include SIRT1 (HPG axis and antioxidant defense), SIRT2 (spindle organisation and oocyte aging), SIRT3 (mitochondrial function and oxidative stress), and SIRT6/7 (DNA repair and genome stability). Recent studies have highlighted the necessity of sirtuin-based treatments to improve fertility outcomes, specifically reproductive aging, polycystic ovary syndrome (PCOS), oxidative stress-related infertility, and ART.

### Class IV HDAC

HDAC11 is the only class IV histone deacetylase that modulates immunological tolerance at the maternal–fetal interface. It also controls the expression of cytokines and immunomodulatory molecules, which are needed for embryo implantation and pregnancy maintenance. HDAC11 particularly stops the production of interleukin-10 (IL-10), which is an essential anti-inflammatory cytokine that enhances maternal immunological tolerance to the semiallogenic foetus. HDAC11 overexpression in dendritic cells reduces IL-10 production, thereby causing a shift in the immune response to a proinflammatory Th1 profile, which can hinder implantation success [11]. Clinical and experimental studies have shown the importance of HDAC11 in reproductive immunology. Women who experience recurrent pregnancy loss (RPL) or implantation failure frequently display abnormal HDAC11 expression in endometrial or decidual tissues, indicating that increased HDAC11 activity may disrupt cytokine balance and uterine receptivity [42]. In mouse models, dysregulated HDAC11 expression affects the uterine cytokine environment, inhibits trophoblast invasion, and disrupts the vascular remodelling required for embryo implantation. Furthermore, targeted inhibition of HDAC11 has been shown in mouse model studies to restore IL-10 levels and improve implantation rates, highlighting its potential as a therapeutic target in reproductive immunopathologies [43]. During gametogenesis in mouse models, HDAC11 regulates meiosis by controlling genes and factors such as AURKB, PCNA, INO80C, and HMBOX1, which are needed for spindle apparatus formation, chromosome segregation, and cell cycle progression and for proper oocyte and spermatocyte maturation. However, similar to other HDACs, balanced HDAC 11 expression is critical, as both deficiency and excessive activation can disrupt meiotic events and chromatin organisation, potentially leading to errors in gamete formation and infertility [44].

Controlled manipulation of HDAC11 activity in preclinical models may thus be a unique method for improving implantation outcomes and reducing immunologically driven pregnancy failure. Future research should focus on better understanding the unique activities of HDAC11 in various reproductive situations and optimising HDAC11-selective inhibitors for therapeutic use.

The localisation, expression patterns, and reproductive functions of different HDAC isoforms are summarised in Table 1.

**Table 1:** Summary of histone deacetylase (HDAC) isoforms: localization, expression, and functional roles in reproductive physiology

HDAC Class / Isoform	Localization / Expression	Reproductive Functions	Findings in Models or Clinical Studies	References
Class I: HDAC1	Nuclear; oocytes, somatic gonadal cells	Maintains chromatin structure, meiosis, follicle viability	HDAC1/2 double knockout: infertility, chromatin disarray, follicular atresia, halted embryogenesis	[6, 20, 21]
Class I: HDAC2	Nuclear; oocytes, granulosa cells	Chromosome segregation, embryo viability	Loss causes segregation errors, reduced embryo survival, isoform-specific controls	[21]
Class I: HDAC3	Nuclear; uterine stromal cells	Decidualization, progesterone signaling, endometrial receptivity	Uterine knockout: implantation failure, hormone signaling disruption	[12]
Class I: HDAC8	Granulosa, testicular cells	Steroidogenesis (StAR, CYP19A1), estradiol synthesis, spermatogonial proliferation	Inhibition impairs follicular maturation, testicular function	[24]
Class IIa: HDAC4, HDAC5, HDAC7, HDAC9	Nucleus/cytoplasm shuttling; various reproductive tissues	Regulate transcription, reproductive tissue development	HDAC4 critical for ovarian function in insects; knockdown causes infertility	[13]
Class IIb: HDAC6, HDAC10	Cytoplasmic; gametes	Tubulin deacetylation, sperm motility, meiotic spindle regulation	HDAC6 knockout: hyperacetylated tubulin, reduced sperm motility under stress; pharmacological inhibition improves gamete quality	[30, 34]
Class III (Sirtuins): SIRT1–7	Nuclear, mitochondrial; oocytes, granulosa cells, testis	Mitochondrial homeostasis, oxidative stress regulation, reproductive ageing, gamete quality	Knockouts show low folliculogenesis, apoptosis, poor fertility; Resveratrol activates SIRT1/SIRT3, improves oocyte and ovarian function	[36, 37]

## Mechanistic Insights into the Role of HDACs

### Role in Spermatogenesis

Spermatogenesis is a process that includes mitosis, meiosis, and spermiogenesis and is controlled epigenetically *via* histone acetylation and deacetylation [45, 46]. In mice, the chromatin-associated tumor suppressor ING2 is required for normal spermatogenesis, and its loss causes male infertility. ING2 is needed for proper HDAC1 accumulation and normal histone acetylation during spermatocyte development. If ING2 is absent, chromatin acetylation becomes abnormal, and germ apoptosis increases, leading to partial p53 signalling. Reduced expression of ING2 is connected to human male infertility, including teratozoospermia and Sertoli-cell-only syndrome, indicating that its role in regulating spermatogenesis is conserved.

Sperm chromatin remodelling and gene transcription require certain histone acetylation sites, such as H3K9, H3K14, H4K5, and H4K16 [33]. HDAC1 and HDAC2 regulate meiotic gene expression, chromatin remodelling, and DNA repair; their deletion causes meiotic arrest and apoptosis in mouse germ cells, emphasising their importance [47]. HDAC3 in mouse spermatocytes functions beyond deacetylation and acts as a transcriptional regulator during meiotic exit by working with SOX30 to control the gene expression required for sperm maturation [48]. Another protein, ZMYND15, functions as a class I and II HDAC to repress gene expression in haploid germ cells; loss of ZMYND15 in mouse models leads to abnormal activation of hundreds of testicular genes and results in azoospermia [49].

Sirtuins (class III HDACs), particularly SIRT1, control metabolism, stress response, and chromatin compaction in testicular cells; their absence in mouse models affects the hypothalamic‒pituitary‒gonadal axis, resulting in stalled spermatogenesis [50]. During spermiogenesis, HDACs modulate histone acetylation, which is needed for chromatin condensation and sperm DNA packaging. HDACs catalyse this process by removing acetyl groups, causing histone eviction and the insertion of transition proteins and protamines. Dysregulation of HDAC activity in mouse models can inhibit the process of histone-to-protamine transition, resulting in chromatin condensation, abnormal sperm morphology, and, ultimately, male infertility [51]. SIRT1 in mouse spermatids competes with MOF acetyltransferase, and SIRT1 inhibition increases the acetylation status of the MOF protein. These findings suggest that SIRT1 plays a regulatory role in the histone-to-protamine transition during spermiogenesis [52].

Alterations in histone modifications in experimental animal models, particularly H3K4me3, H3K9me3, H3K27me3, and H4 acetylation, cause interference in sperm chromatin remodelling, leading to DNA damage and fertilisation problems. Proper DNA packaging, which is controlled by chromatin-remodelling proteins such as SWI/SNF, ISW1, and MI-2, is necessary for sperm function, and defects in these proteins cause abnormal sperm morphology and movement. Additionally, miRNAs such as miR-34 play an influential role in sperm maturation, and their imbalance has been linked to spermatogenic disorders [53]. In cryptorchidism, elevated levels of HDAC1, HDAC2, and HDAC3 disrupt chromatin structure and are strongly linked to infertility risk, which may continue across generations owing to transgenerational epigenetic inheritance [54].

Pharmacological HDAC inhibitors, such as trichostatin A (TSA), can reversibly slow down meiosis in mouse testicular tissue, increase spermatocyte death, and cause temporary infertility [55]. In asthenozoospermic (low-motility) sperm, HDAC6 expression is reduced along with acetyl alpha-tubulin levels. The HDAC6-specific inhibitor tubastatin A can impair sperm motility and microtubule stability, suggesting that threshold levels of microtubule acetylation/deacetylation are critical for proper sperm flagellar dynamicity and that abnormal HDAC6 expression and tubulin acetylation status may contribute to male infertility [56].

Importantly, HDACs regulate the metabolism of seminal plasma, with changes in HDAC activity associated with variations in sperm motility and energy availability [57]. SIRT1 and SIRT3 levels in seminal plasma are negatively correlated with oxidative stress and DNA fragmentation in asthenoteratozoospermia. Low SIRT1 and SIRT3 protein levels are correlated with abnormal sperm morphology and motility, suggesting that these sirtuins play protective roles in the maintenance of sperm quality [58].

Chromatin integrity tests, such as terminal deoxynucleotidyl transferase dUTP nick-end labelling (TUNEL) and sperm chromatin dispersion (SCD), are being employed clinically to identify DNA fragmentation associated with aberrant HDAC function [59].

**Fig. 2:**
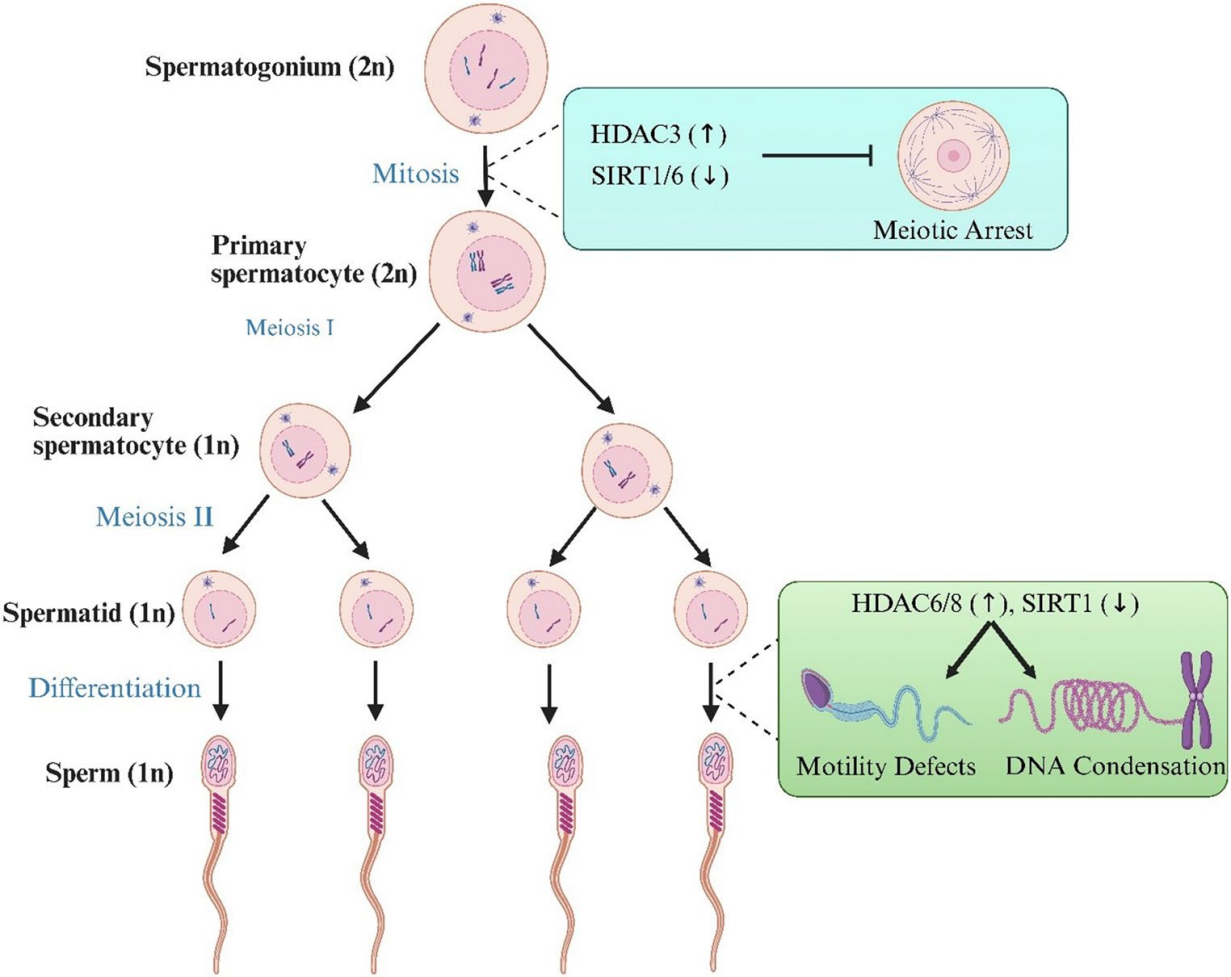
Altered HDAC and SIRT expression disrupts key stages of spermatogenesis, leading to meiotic arrest, impaired DNA condensation, and sperm motility defects

Collectively, altered HDAC and sirtuin expression disrupts multiple stages of spermatogenesis, resulting in meiotic arrest, defective chromatin condensation, and impaired sperm motility (Fig. 2).

### Role in Oogenesis and Folliculogenesis

HDACs strongly control chromatin dynamics during oocyte development and folliculogenesis. A study by Szalai et al. [60] revealed a significant decrease in HDAC1 and HDAC2 expression in women of advanced reproductive age and those with low ovarian reserve (LOR) compared with younger women with normal ovarian reserve (NOR). These lower HDAC levels were closely linked to the quantity of recovered and mature (MII) oocytes, suggesting that HDAC1 and HDAC2 are necessary for optimal folliculogenesis and oocyte maturation. By changing chromatin structure through histone deacetylation, HDACs mechanistically control gene expression. The downregulation of these genes most likely interferes with the transcriptional processes necessary for the formation of the cumulus–oocyte complex [60]. During the luteinising hormone (LH) surge in mouse ovaries, HDAC2 is essential in GCs because its phosphorylation causes histone deacetylation and transcriptional reprogramming, which are required for ovulation, cumulus growth, and the expression of ovulatory genes such as Ereg and Star. HDAC3 overexpression in mouse GCs hinders oocyte maturation by inhibiting the increase in normal histone acetylation, which can be reversed with HDAC3 inhibitors, suggesting therapeutic potential in female infertility [61].

Sirtuins, particularly SIRT1, influence oocyte quality by controlling mitochondrial function and redox equilibrium. SIRT2 plays a critical role in oocyte meiosis. SIRT2 depletion in mouse oocytes causes severe spindle defects and chromosomal disorganisation, with 35.5% of the SIRT2-knockdown oocytes showing abnormal spindles and chromosomes compared with 9.6% of the control oocytes. SIRT2 modulates the acetylation status of both histone H4K16 and α-tubulin, which mediate defective phenotypes by influencing microtubule dynamics and kinetochore function. Critically, lower SIRT2 protein levels in oocytes from aged mice were observed, and SIRT2 overexpression ameliorated maternal age-associated meiotic defects, reducing abnormalities from 33.2% in old oocytes to 12.7% in old oocytes with SIRT2 overexpression [62]. Transgenic SIRT2 (SIRT2-Tg) overexpression prolongs female fertility with improved oocyte quality, ovulation rates, and pregnancies during aging. SIRT2-Tg mice maintain pregnancies at late reproductive ages (14–16 months), demonstrating sustained improvement in fertility with increasing age [63]. SIRT5 is located predominantly at the periphery of the meiotic spindle and is enriched on chromosomes during mouse oocyte maturation. Mechanistically, SIRT5 plays dual roles in maintaining mitochondrial homeostasis: (1) regulating Parkin-dependent mitophagy to prevent excessive mitochondrial clearance and (2) preserving the NADPH/GSH antioxidant system to ensure redox balance. SIRT5 inhibition causes significant meiotic defects, including disrupted spindle arrangement and chromosome misalignment linked with increased histone acetylation, that impair kinetochore–microtubule attachment [64]. SIRT6 in porcine oocytes essentially functions to drive oocyte meiotic maturation, as it helps maintain normal spindle/chromosome structure formation, actin formation, and cumulus cell distribution. The inhibition of SIRT6 expression significantly increases the levels of reactive oxygen species (ROS) signalling, leads to DNA damage and induces apoptosis, leading to oocyte meiotic defects through the impairment of polar body extrusion and cumulus cell expansion in porcine oocytes [65].

Other HDACs, including HDAC6 and HDAC11, are dynamically expressed during follicular growth and maturation and regulate oocyte competence in mouse models [8]. Reduced HAT1 expression in GCs disturbs oocyte maturation in aged mice, thereby highlighting the importance of the acetylation/deacetylation balance regulated by HATs and HDACs [66]. Abnormal HDAC expression, such as high HDAC3 levels in GCs, prevents oocyte maturation in mouse models and effective ovulation, yet targeted HDAC3 inhibitors can restore this phenomenon, indicating promising fertility-restoration techniques [61].

HDACs collaborate with methyltransferases and microRNAs to regulate follicular development and endocrine signalling. In conditions such as PCOS and premature ovarian insufficiency (POI), dysregulated HDAC activity interferes with these pathways, contributing to infertility while also presenting potential epigenetic therapy targets [67].

**Fig. 3:**
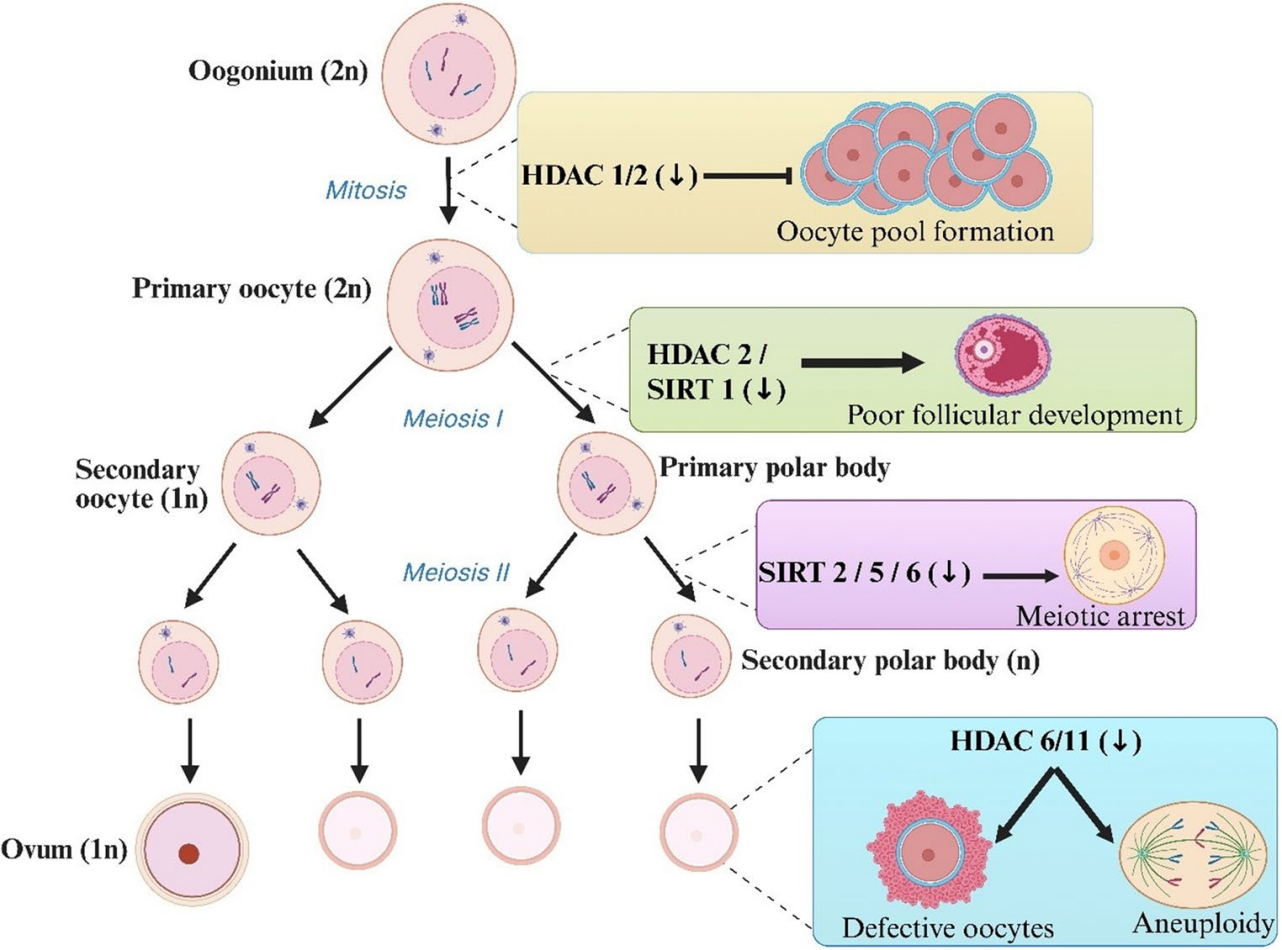
Altered HDAC and SIRT expression disrupts key stages of oogenesis, leading to impaired follicular development, meiotic arrest, and defective oocyte formation

These findings highlight the critical role of HDAC and sirtuin dysregulation in oogenesis and folliculogenesis, leading to impaired follicular development and defective oocyte maturation (Fig. 3).

### Early Embryonic Development and Epigenetic Reprogramming

During early development stages, HDACs control the chromatin remodelling and epigenetic reprogramming required for the maternal-to-zygotic transition and early cell fate determination [68, 69]. Wang et al. [70] demonstrated that HDAC activity is very much required for preventing the premature expression of developmental genes during zygotic genome activation. Their study revealed that HDAC inhibition from 8 to 28 h post-fertilisation in mouse embryos causes embryo developmental arrest at the 2-cell stage [70]. HDAC1 and HDAC2 control global histone acetylation levels, and their absence in mouse embryos causes abnormal ZGA, increased apoptosis, and developmental arrest before blastocyst formation [45, 71]. English et al. [72] revealed that rapid HDAC1 degradation causes changes in histone acetylation within 2 h and affects pluripotency-associated gene networks in mouse embryonic stem cells, including the downregulation of superenhancer-associated genes [72]. HDAC1 progressively binds to the *Xenopus* zygotic genome beginning at the mid-blastula stage and maintains lineage integrity by preserving histone hypoacetylation at inactive genes while participating in dynamic acetylation–deacetylation cycles at active genes [73]. HDAC1 and HDAC2 are essential for syncytiotrophoblast development, with the deacetylation of multiple core histone residues associated with human trophoblast differentiation [74]. Duan et al. [75] reported that HDAC inhibition upregulates placental P-glycoprotein expression in trophoblast cells, with HDAC1/2 involved in regulating drug transplacental transfer [75].

HDAC inhibition, such as by TSA therapy, increases histone acetylation and can temporarily improve nuclear reprogramming, as shown in mouse somatic cell nuclear transfer models, by erasing somatic chromatin memory and increasing blastocyst formation rates [76]. Pan-HDACi treatment directly reprogrammed murine embryonic stem cells toward a 2-cell embryo state, significantly increasing the population of 2 C-like cells in ESC cultures. These findings suggest that HDAC inhibition can facilitate early embryonic developmental transitions [77].

However, severe or prolonged HDAC inhibition can impair chromatin condensation and gene silencing, resulting in poor embryo quality and unsuccessful developmental transitions [78].

### Uterine Receptivity and Implantation

The balance between histone acetylation and HDAC activity is critical for uterine preparation for implantation [79]. During decidualisation, HDAC2 downregulation in human endometrial stromal cells (EnSCs) in vitro increased the expression of genes that are required for successful embryo implantation. Errors in this process might result in infertility or implantation failure [80]. Endometriosis mouse models and human samples exhibit reduced HDAC3 levels, resulting in decidualisation abnormalities, fibrosis, and poor progesterone signalling, all of which lead to infertility. Restoring HDAC3 function or regulating related epigenetic variables in preclinical models may provide new treatments for endometriosis-induced infertility [12].

HDAC inhibitors (e.g., SAHA and TSA) increase the expression of glycodelin, a modulator of embryo–endometrial attachment, in human endometrial cells in vitro, suggesting their therapeutic potential for enhancing implantation and treating endometrial diseases [67]. In contrast, high HDAC activity caused by inflammation, such as LPS exposure in mouse models, decreases ovarian estradiol production through chromatin remodelling, which can be reversed by HDAC inhibition [55]. HDACs may contribute to ovarian dysfunction, such as that associated with PCOS and POI, by impairing follicular development, steroidogenesis, and hormone receptor signalling. Genetic research, such as on SNP correlations in HDAC1 in South Indian women, highlights the relevance of HDACs in real-world infertility risk [81].

## HDACs and Assisted Reproductive Technologies (ART)

HDACs have emerged as important modulators in ART in experimental and preclinical models, as they regulate chromatin architecture, epigenetic reprogramming, and embryonic development.

HDAC inhibitors, such as TSA and Scriptaid, have been shown to improve histone acetylation in somatic cell nuclear transfer (SCNT) and IVF models across multiple mammalian species, opening the chromatin structure and allowing the transcriptional activation required for early embryonic development [82].

For example, treating pig SCNT embryos with scriptaid significantly increased blastocyst formation rates, increased the similarity of histone acetylation patterns to those of IVF embryos, and improved overall cloning efficiency [83, 84]. Similar favourable effects have been observed for TSA in various species, including mice and rabbits, where increased acetylation has been associated with effective zygotic genome activation and improved embryo viability [76]. HDAC6 regulates histone acetylation in mouse oocyte vitrification models and preserves developmental competence during cryopreservation, which is essential for improving oocyte vitrification (OV) efficiency. The postvitrification survival, cleavage, and blastocyst formation rates were markedly lower in oocytes with low HDAC6 levels. Mechanistically, miR-762 binds to the 3′-UTR of HDAC6 and posttranscriptionally downregulates it, preventing its translation without changing its mRNA level. HDAC6 overexpression or suppression of miR-762 increased HDAC6 protein levels and greatly increased the number of cryopreserved oocytes. These results imply that fertility preservation techniques and oocyte cryopreservation procedures may be improved in preclinical cryopreservation models by targeting the miR-762–HDAC6 axis [85]. Elevated serum and follicular fluid SIRT levels are associated with improved fertility parameters. Yao et al. [86] demonstrated that serum and follicular fluid SIRT1 and SIRT2 protein levels are correlated with age, anti-Mullerian hormone (AMH) levels, the antral follicle count (AFC), and oocyte number, suggesting that these sirtuins have predictive value for assisted reproductive outcomes [86].

A study by Yang et al. [87] revealed that male infertility, namely, asthenozoospermia, a disorder characterised by low sperm motility, is affected by the HDAC6 inhibitor scriptaid. In both mouse IVF and human intracytoplasmic sperm injection (ICSI) models, scriptaid-treated sperm increased the rates of fertilisation and embryo development. To influence sperm motility and structural integrity, scriptaid works mechanistically by modifying protein acetylation and increasing tyrosine phosphorylation. Important sperm molecules linked to energy and motility have also changed, suggesting that this technique is a viable tool for enhancing the quality of sperm and the success of fertilisation in ART [87].

The HDAC inhibitor scriptaid greatly improves blastocyst formation and developmental competence in ovine SCNT embryos by increasing histone acetylation and nuclear reprogramming efficiency [88]. These findings were verified in cashmere goats, where scriptaid increased donor cell pluripotency by upregulating NANOG expression, validating the species-wide effects of HDAC inhibition on large animal cloning [89].

Similarly, HDAC inhibition restored progesterone receptor (PR) expression and the expression of downstream target genes (FOXO1, p21, AREG, and PAEP) in human endometrial cells, suggesting therapeutic potential for improving ART implantation rates [90]. HDACs have different regulatory effects on ovarian dysfunction, particularly in the context of PCOS. HDAC5 overexpression in mouse PCOS models reduces oxidative stress and abnormal angiogenesis, improving ovarian morphology and function [91], whereas HDAC1 restoration in mouse granulosa cells reduces pyroptosis *via* the H19/miR-29a-3p/NLRP3 axis, reducing inflammation and hormone imbalance [92]. Integrating artificial intelligence (AI)-based embryo selection with epigenetic profiling and HDAC activity evaluation has been recommended as a game-changing next step for personalised ART [93].

Among pharmacological modulators, scriptaid, TSA, valproic acid (VPA), sodium butyrate, and HDAC3-selective inhibitors have demonstrated the most consistent benefits in improving nuclear transfer efficiency and in vitro maturation outcomes, with blastocyst formation increasing by up to 60–79% relative to that of untreated SCNT or IVF control embryos across various mammalian models [76, 83, 88] Collectively, these data show that targeted HDAC modulation, particularly isoform-selective inhibition, holds enormous promise for increasing ART effectiveness and embryo quality and for resolving ovarian and endometrial diseases *via* precise epigenetic reprogramming.

These findings indicate that HDAC inhibition can partially improve nuclear reprogramming efficiency and blastocyst development in preclinical SCNT and IVF animal models. Studies suggest that compared with control conditions, TSA treatment during the extended culture of oocytes in vitro resulted in significantly reduced fertilisation and blastocyst formation, thereby implying that TSA had a negative effect on early embryonic development in this mouse model after continuous oocyte culture [94]. These findings also suggest that certain ART-related manipulations and extended in vitro culture conditions may alter histone acetylation and methylation patterns in animal models.

## Environmental and Lifestyle Factors Influencing HDACs in Infertility

Environmental factors such as endocrine-disrupting chemicals (EDCs), heavy metals and lifestyle can influence HDAC expression and activity, impacting reproductive results [95]. In mouse models, bisphenol A (BPA) and phthalate exposure reduce HDAC1/2 activity in both testes and ovaries, resulting in altered spermatogenesis and folliculogenesis, with some effects lasting for generations [96]. Similarly, alterations in histone modifications associated with exposure to heavy metals such as lead and cadmium have been linked to decreases in sperm motility and count [97]. A study by Besong et al. [98] revealed that arsenic-induced male infertility causes increased activity of class I HDACs in rat testes, which represses genes essential for spermatogenesis and induces oxidative-inflammatory damage. Acetate inhibits class I HDACs and helps reduce harmful signalling in reproductive tissues [98]. Stress and smoking alter HDAC2 expression and can cause impairment of uterine function and embryo implantatio [98, 100]. Psychological stress also activates the hypothalamic‒pituitary‒adrenal (HPA) axis, increasing cortisol and corticotropin-releasing hormone (CRH) levels, which suppress gonadotropin release *via* hypothalamic HDAC dysregulation, whereas decreased histone acetylation (H3K9ac, H3K14ac) in reproductive regulatory regions impairs fertility-related gene expression and promotes granulosa apoptosis, ultimately lowering conception rates in IVF patients [101]. Furthermore, recent research has revealed that EDCs such as ddchlorodiphenyldichloroethylene (DDE), hexachlorobenzene (HCB), and BPA are associated with the upregulation of class I HDACs (such as HDAC1, HDAC2, and HDAC3). This upregulation leads to increased histone deacetylation activity, causing aberrant chromatin remodelling and suppression of gene expression necessary for normal reproductive processes, leading to decreased fertility rates, fewer high-quality embryos, an increased risk of miscarriage, and lower ART success [102]. In rodent heat stress models, elevated testicular temperature caused by environmental or occupational heat stress induces HDAC-mediated germ apoptosis, oxidative damage, chromosomal defects, and impaired spermiogenesis [103, 104]. In experimental models, oxidative stress further worsens infertility by activating HDAC-dependent silencing of the expression of genes encoding antioxidants such as SOD, catalase, and GPx, whereas reduced sirtuin activity weakens mitochondrial ROS defense and damages sperm DNA [105]. In contrast, experimental and clinical studies have shown that antioxidant supplementation can restore HDAC-regulated antioxidant gene expression and improve fertility parameters [106].

Environmental chemicals such as triclosan, BPA, DEHP, and PFAS disrupt HDAC activity and histone modifications, leading to hormonal imbalance, reduced endometrial receptivity, and even transgenerational reproductive toxicity in animal and epidemiological studies [107, 108]. Nutritional interventions, especially dietary restriction, increase histone acetylation through HDAC inhibition and sirtuin activation, which can improve reproductive capacity with age in experimental models [7, 109]. Chronic inflammation and oxidative stress also increase HDAC activity, reducing the expression of pro-survival genes and impairing mitochondrial function [110].

Furthermore, age and developmental stage influence HDAC sensitivity to stresses such as heat, with spermatogenesis being more sensitive than oogenesis [111].

**Fig. 4:**
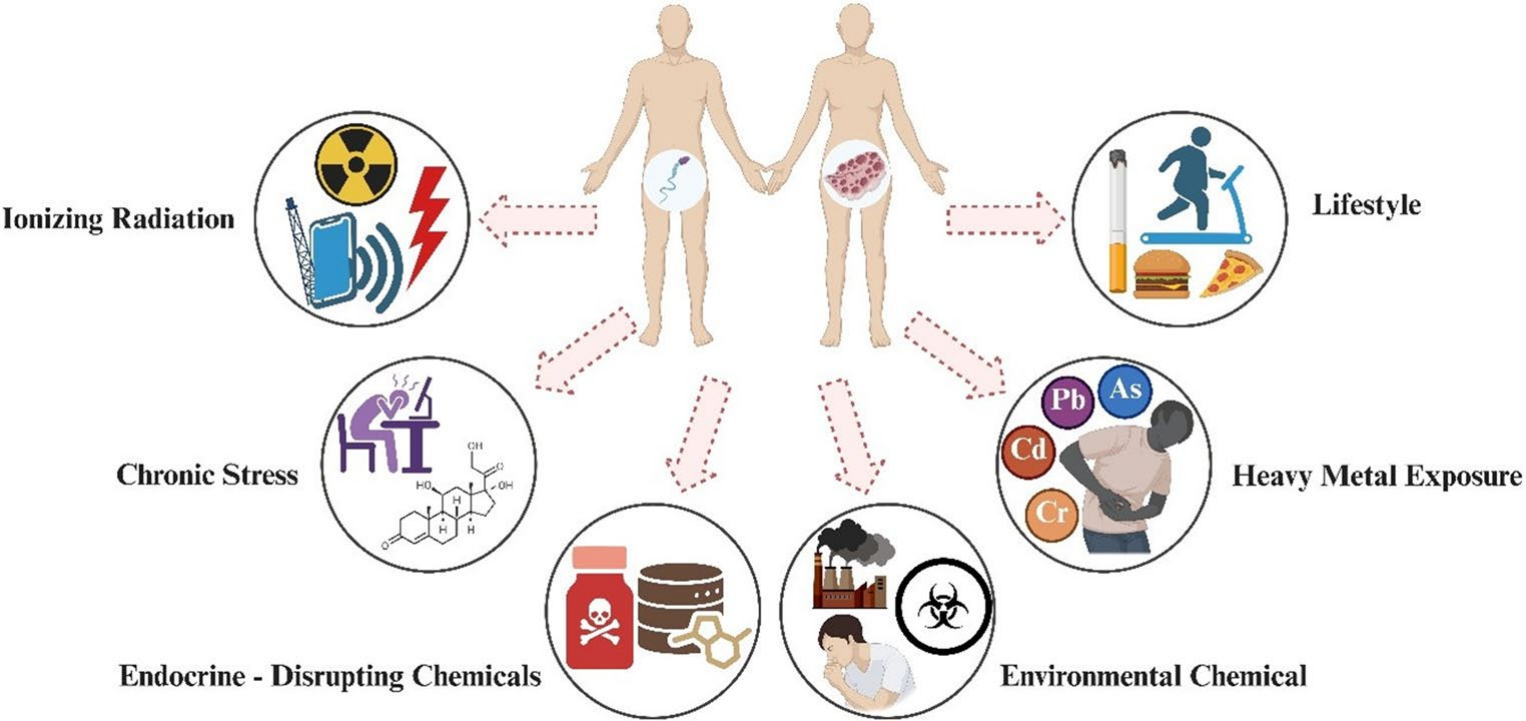
Diagram illustrating environmental and lifestyle factors that modulate HDAC activity and contribute to infertility

Collectively, these studies show that psychological stress, environmental heat, oxidative imbalance, and chemical exposure contribute to fertility loss *via* HDAC dysregulation, whereas dietary and antioxidant interventions targeting HDAC pathways provide practical, low-cost strategies for prevention and treatment (Fig. 4).

## Therapeutic Potential of HDAC Modulation in Infertility

HDACs play crucial roles in regulating fertility-related processes such as gametogenesis, oocyte maturation, endometrial receptivity, and early embryonic development. Endometriosis, PCOS and poor spermatogenesis are associated with abnormal HDAC activity or expression. As a result, HDACs have emerged as promising therapeutic targets in reproductive medicine. Several HDAC inhibitors (HDACis) that were originally developed for cancer therapy, such as VPA, which increases the reprogramming efficiency of primary human fibroblasts into induced pluripotent stem cells, have shown potential for improving reproductive outcomes, suggesting that epigenetic therapy may aid in correcting infertility of metabolic or age-related origin [112]. Broad-spectrum HDAC inhibitors such as TSA and VPA have demonstrated potential for improving oocyte quality, fostering embryonic development, and increasing implantation rates in animal models, whereas isoform-specific inhibitors, including tubastatin A (HDAC6-selective) and romidepsin (HDAC1/2), provide more focused strategies with less systemic toxicity and off-target consequences. Given that particular HDAC isoforms, such as HDAC3 and HDAC6, are crucial for spermatid maturation and sperm motility, these compounds may have therapeutic potential in the treatment of male infertility [48].

However, their practical application presents difficulties because of the extensive regulatory roles of HDACs, potential off-target effects, and the need for precise, stage-specific interventions. Genomic and nongenomic profiling could enable personalised HDAC inhibitor therapy based on individual HDAC expression, such as the use of class I inhibitors in endometriosis patients with high HDAC1/2 levels [113]. Advances in single-cell sequencing, epigenetic mapping, and noninvasive biomarker identification could improve patient selection and monitoring. Combining HDACis with hormonal or assisted reproductive therapy may also increase efficacy while minimising systemic effects [114]. Future research should concentrate on designing HDAC modulators tailored to reproductive tissues, ensuring long-term safety, and standardising clinical methods. Promising candidates, such as scriptaid, HDACi-14, and HDACi-79, have shown better embryo development and stem cell derivation in animal models, confirming that HDAC inhibition is a viable experimental technique for increasing fertility [114]. Agents that activate SIRT1 and SIRT3 improve ovarian function and delay aging-related fertility decline [36]. Targeting HDACs, particularly HDAC5, may be an effective technique for treating infertility caused by metabolic and endocrine disorders such as diabetes. Type 2 diabetes mellitus (T2DM) is linked to increased HDAC5 expression in the brain and ovary, which disrupts the hypothalamic‒pituitary‒ovarian (HPO) axis and impairs ovarian hormone control and fertility. Acetate inhibits HDAC5 activity, helping to restore normal histone acetylation and gene expression in the reproductive system. In diabetic animals, this improves insulin sensitivity and helps preserve ovarian function [115].

Combination therapies that target both oxidative stress and epigenetic changes are being explored. In a PCOS rat model, acetate improved ovarian function by reversing abnormal overexpression of HDACs, especially HDAC1 and HDAC3, in GCs [116]. This abnormal HDAC activity contributes to granulosa cell death, disrupted steroid hormone production, oxidative stress, and poor follicle growth, all of which are linked to PCOS-related infertility.

Acetate therapy normalises histone acetylation, improves the ovarian hormone balance, decreases oxidative stress, and restores follicle maturation and ovulation [116]. Another study by Besong et al. [98] revealed that the administration of sodium acetate to Wistar rats subjected to testicular ischemia/reperfusion injury reduces the increase in HDAC class I and II activity caused by ischemia/reperfusion, thereby restoring normal histone acetylation levels. This epigenetic modulation helps suppress oxidative stress and inflammation in testicular tissue, maintaining the integrity of spermatogenic cells and steroidogenic function. Consequently, by acting as a HDACi, sodium acetate preserves sperm quality (count, motility, viability, morphology) and testicular histoarchitecture, protecting fertility after injury [98].

Moreover, the role of HDACs in contraceptive therapeutics is under investigation. Hong et al. [117] demonstrated that oral administration of the class I HDACi MS-275 reversibly blocks spermatogenesis and fertility in mice without affecting libido. This HDACi targets the SMRT-retinoic acid receptor-HDAC complex, providing a promising nonhormonal male contraceptive approach with complete fertility restoration within 60 days of treatment cessation [117].

## Limitations

Despite increasing interest in the role of HDACs in reproductive biology, several limitations should be acknowledged. Much of the available research relies on experimental animal models rather than human clinical studies. For instance, studies investigating the therapeutic effects of HDAC modulation on ovarian function and PCOS have primarily been conducted in rat models, in which acetate treatment was shown to restore histone acetylation and improve ovarian function [116]. Similarly, the protective effects of sodium acetate on spermatogenesis and testicular histoarchitecture were demonstrated in Wistar rats subjected to testicular ischaemia‒reperfusion injury, highlighting the reliance on animal systems for mechanistic insights [98]. In addition, several studies assessing the role of HDAC inhibitors in reproductive biology focus mainly on short-term development outcomes. For example, HDAC inhibition using compounds such as scriptaid has been reported to improve embryo development and stem cell derivation in experimental models, yet long-term reproductive safety and transgenerational effects have not been evaluated [114]. Furthermore, although HDACs have been implicated in spermatogenesis and male infertility, many studies have focused on general HDAC activity rather than the precise roles of individual HDAC isoforms, making determining isoform-specific mechanisms difficult [48]. Finally, while HDAC expression is associated with reproductive disorders such as endometriosis, further clinical validation is needed before HDAC-targeted therapies can be translated into routine reproductive medicine [113]. Collectively, these limitations highlight the need for well-designed human studies, long-term clinical investigations, and integrative multiomics approaches to better define the clinical relevance and therapeutic potential of HDAC-based strategies in infertility.

## Future Perspectives

HDACs have emerged as crucial epigenetic regulators of reproductive function, controlling a wide range of cellular activities essential for both male and female fertility. This study highlights the intricate, stage-specific roles of HDACs in spermatogenesis, oocyte maturation, embryogenesis, and endometrial receptivity.

Various HDAC isoforms constantly regulate histone and nonhistone protein acetylation, allowing the precise transcriptional control and chromatin remodelling required for gametogenesis and reproductive tissue homeostasis. Male spermatid elongation, flagellar assembly, and DNA repair are dependent on HDAC1, HDAC3, and HDAC6. Deficiencies in these activities can result in impaired fertilisation capacity, DNA fragmentation, and poor motility [48]. Similarly, in females, HDACs control critical processes such as folliculogenesis, steroidogenesis, and meiotic arrest. PCOS and endometriosis are two disorders associated with infertility that are triggered by HDAC expression and activity, which change the epigenetic environment of oocytes and endometrial cells [113].

The ability of HDACs to affect histone acetylation patterns and larger gene regulatory networks by deacetylating transcription factors and signalling proteins is what makes them so fascinating. Because of their pleiotropic effects, HDACs have been shown to be both biomarkers and possible targets for infertility treatments. However, to prevent systemic toxicity, any therapeutic modification of HDAC activity needs to be extremely selective and tissue specific owing to its widespread expression and diverse cellular roles.

Abnormal HDAC expression in reproductive disorders emphasises the necessity of integrating epigenetic profiling into fertility diagnostics from a therapeutic standpoint. A noninvasive biomarker for the sperm epigenome or endometrial biopsy analysis, for example, could indicate altered HDAC activity. Promising candidates for repurposing in reproductive medicine are selective HDACiss, which are currently being studied for neurological diseases and cancer. Although the long-term safety and effectiveness of HDACis in humans have not yet been confirmed, animal models have demonstrated that they can improve implantation rates and increase embryonic growth [118].

## Conclusion

Mapping the isoform-specific activities of HDACs in reproductive organs throughout developmental stages and under different hormonal conditions is critical for completely understanding their contribution to fertility. Single-cell transcriptomics and spatial epigenomics provide high-resolution insights into how HDACs influence cell fate decisions in gametes and early embryos. In parallel, investigating how lifestyle and environmental factors influence HDAC activity will aid in understanding the link between modern stressors such as aging, obesity, and endocrine-disrupting chemicals and the global decline in fertility. Collectively, these findings show that HDACs serve as major epigenetic axes that integrate transcription, signalling pathways, and chromatin architecture to maintain reproductive competence. With advances in reproductive epigenetics, the diagnostic and therapeutic potential of targeting HDACs may provide novel opportunities for infertility treatment. However, since epigenetic changes are heritable and might have transgenerational consequences, such innovations must be supported by rigorous preclinical validation and careful ethical consideration.

## Data Availability

No data was used for the research described in the article.

## Abbreviations

ART: Assisted reproductive technology
GCs: Granulosa cells
H3K9ac: Acetylated lysine 9 on histone H3
H4K16ac: Acetylated lysine 16 on histone H4
HDAC: Histone deacetylase
HDACi: Histone deacetylase inhibitor
IVF: In vitro fertilization
PCOS: Polycystic ovary syndrome
PR: Progesterone receptor
ROS: Reactive oxygen species
SCNT: Somatic cell nuclear transfer
SIRT: Sirtuin
TSA: Trichostatin A
VPA: Valproic acid
ZGA: Zygotic genome activation

## Declarations

### **Generative AI and AI-Assisted Technologies in the Writing Process **

During the preparation of this work, the author(s) used Quillbot to paraphrase. After using this tool/service, the author(s) reviewed and edited the content as needed and take(s) full responsibility for the content of the publication.
